# Visible Light Driven Photoanodes for Water Oxidation Based on Novel r-GO/β-Cu_2_V_2_O_7_/TiO_2_ Nanorods Composites

**DOI:** 10.3390/nano8070544

**Published:** 2018-07-18

**Authors:** Shuang Shuang, Leonardo Girardi, Gian Andrea Rizzi, Andrea Sartorel, Carla Marega, Zhengjun Zhang, Gaetano Granozzi

**Affiliations:** 1 University of Padova and INSTM Unit, via Marzolo 1, 35121 Padova, Italy; shuangshuang_buct@163.com (S.S.); leonardo.girardi@phd.unipd.it (L.G.); andrea.sartorel@unipd.it (A.S.); carla.marega@unipd.it (C.M.); gaetano.granozzi@unipd.it (G.G.); 2 State Key Laboratory of New Ceramics and Fine Processing, School of Materials Science and Engineering, Tsinghua University, Beijing 100084, China; 3 Key Laboratory of Advanced Materials (MOE), School of Materials Science and Engineering, Tsinghua University, Beijing 100084, China; zjzhang@mail.tsinghua.edu.cn

**Keywords:** copper vanadate, photoanode, water splitting, graphene oxide

## Abstract

This paper describes the preparation and the photoelectrochemical performances of visible light driven photoanodes based on novel r-GO/β-Cu_2_V_2_O_7_/TiO_2_ nanorods/composites. β-Cu_2_V_2_O_7_ was deposited on both fluorine doped tin oxide (FTO) and TiO_2_ nanorods (NRs)/FTO by a fast and convenient Aerosol Assisted Spray Pyrolysis (AASP) procedure. Ethylenediamine (EN), ammonia and citric acid (CA) were tested as ligands for Cu^2+^ ions in the aerosol precursors solution. The best-performing deposits, in terms of photocurrent density, were obtained when NH_3_ was used as ligand. When β-Cu_2_V_2_O_7_ was deposited on the TiO_2_ NRs a good improvement in the durability of the photoanode was obtained, compared with pure β-Cu_2_V_2_O_7_ on FTO. A further remarkable improvement in durability and photocurrent density was obtained upon addition, by electrophoretic deposition, of reduced graphene oxide (r-GO) flakes on the β-Cu_2_V_2_O_7_/TiO_2_ composite material. The samples were characterized by X-ray Photoelectron Spectroscopy (XPS), Raman, High Resolution Transmission Electron Microscopy (HR-TEM), Scanning Electron Microscopy (SEM), Wide Angle X-ray Diffraction (WAXD) and UV–Vis spectroscopies. The photoelectrochemical (PEC) performances of β-Cu_2_V_2_O_7_ on FTO, β-Cu_2_V_2_O_7_/TiO_2_ and r-GO/β-Cu_2_V_2_O_7_/TiO_2_ were tested in visible light by linear voltammetry and Electrochemical Impedance Spectroscopy (EIS) measurements.

## 1. Introduction

The hydrogen economy is a new intriguing sustainable scenario, and it is expected that sooner or later it is going to replace the hydrocarbon economy [1]. With this perspective, a worldwide goal is to provide sustainable and convenient methods to prepare hydrogen fuel. Among them, water splitting (WS), by exploiting the energy of sun, is the most appealing, and many different approaches are currently being investigated [2]. The advantage of the photoelectrochemical (PEC) approach, compared to the standard photocatalytic one, is that an external potential is used to facilitate the WS process. The intrinsic simplicity of PEC, which combines the light absorber and the energy converter into a single device capable to store solar energy into chemical bonds, is rather evident. For these reasons, numerous studies have been performed to fabricate semiconducting nanomaterials with enhanced PEC properties under visible light. In particular, many efforts have been done on developing materials for photoanodes, where the kinetically hindered Oxygen Evolution Reaction (OER) is occurring. Their final performance depends upon the electrocatalysts stability against oxidation [3] and on their intrinsic band energetics [4].

Metal oxide semiconductors are promising photoanode materials because of their relative stability to oxidative photo-corrosion and their low-cost. Hematite, (α-Fe_2_O_3_), has been identified as an efficient photoanode material characterized by a sufficiently low band gap of 1.9–2.0 eV, to be used as “top” electrode in a “tandem” WS device [5]. Nevertheless, this material has some shortcomings, such as a short carrier diffusion length, a significant recombination and indirect absorption. During the last few years, other multicomponent oxides have been suggested as possible active materials for the construction of photoanodes. Among them, ZnFe_2_O_4_, CuWO_4_ CuW_1−*x*_Mo*_x_*O_4_ and especially Cu-vanadates are particularly studied. As with many transition metal (TM) vanadates, Cu-vanadates are characterized by different phases where the Cu/V ratio has a quite large variability. The performances of these phases, together with their PEC stability, were tested in a very comprehensive paper by Gregoire et al. [6]. In their study it was shown that sputter-deposited phases having lower Cu/V ratios are less stable, in borate buffer solution (pH = 9.2), than other phases with higher Cu/V ratio, (e.g., γ-Cu_3_V_2_O_8_ and Cu_11_V_6_O_26_). The γ-Cu_3_V_2_O_8_ based photoanodes, prepared by sol-gel method were also recently studied by Neale et al. [7]. The results of these studies were that the V-rich phases suffered from V loss and a consequent decay in the PEC properties, while the higher stability of Cu rich phases was attributed to a self-passivating mechanism that led to the formation of Cu^+^ and Cu^2+^ oxides on the vanadate surface. In a successive study, again by Gregoire et al. [4], a library of Cu-vanadates thin films with variable stoichiometry was prepared by a fast and convenient ink-jet printing procedure and, again, it resulted that both α-CuV_2_O_6_ and α-Cu_2_V_2_O_7_ are highly active and stable photocatalysts in a borate buffer solution, while β-Cu_2_V_2_O_7_ demonstrated a high photoelectroactivity in the presence of ferri/ferrocyanide redox couple at pH = 13. The abovementioned phases, α-CuV_2_O_6_ and β-Cu_2_V_2_O_7_ were also deposited by a simple drop casting method on fluorine doped tin oxide (FTO) glass and their PEC properties characterized by Mullins et al. [8]. This study showed that the V rich α-CuV_2_O_6_ phase is the one showing the highest photocurrent, although both phases were characterized by a short diffusion length for holes and required the addition of a hole scavenger like Na_2_SO_3_ to improve the photocurrent density. Finally, in a more recent paper by Sharp et al. [9] it was shown that, although Cu-rich phases show higher absorption and charge separation, these phases also present a higher surface recombination rate. Therefore, considering that Cu-rich phases are the ones showing the higher stability in borate buffer solutions, good charge separation and higher absorption and that, on the other hand, V rich phases seem to be those characterized by the higher photocurrents density, we decided to concentrate our attention on the β-Cu_2_V_2_O_7_ phase, that appeared to be a good compromise between durability and PEC performances. The idea of increasing the adhesion between the substrate and the vanadate particles, in order to improve the durability in the electrolyte solution, led us to think about the use of a high surface area substrate like TiO_2_ nanorods (NRs) on FTO [10], to grow this n-type semiconductor. Moreover, the addition of graphene oxide (GO) flakes, could lead to the formation of a composite material with interesting PEC proprieties in term of durability and photocurrent density. In fact, quite recently, composite systems like BiVO_4_/TiO_2_ and V_2_O_5_/BiVO_4_/TiO_2_ were prepared by hydrothermal synthesis and, although not used as active material in photoanodes, showed superior photocatalytic performances in the degradation of organics caused by an upward shift of V_2_O_5_ and BiVO_4_ conduction bands with respect to TiO_2_ with formation of an n-n junction [11,12]. A similar approach was also used by Chen and coworkers [13] where TiO_2_ NRs were decorated by Fe_2_O_3_ grown on preformed TiO_2_ NRs obtained by a simple hydrothermal synthesis. With respect to the effect of the addition of GO flakes, it is useful to remind that GO and especially partial reduced GO (r-GO), are considered as a good support for nanostructures because of their carrier mobility [14], large specific area and high optical transmittance [15,16]. Moreover, when TiO_2_ nanostructures are combined with GO or r-GO they usually can shuttle and store more electrons due to the formation of many p-n nanojunctions with r-GO, a p-type semiconductor [17,18,19].

In this study, we describe the preparation and evaluation of the PEC performances of a visible light driven photoanode based on a novel composite material consisting on β-Cu_2_V_2_O_7_ nanoparticles deposited on TiO_2_ NRs followed by the addition of r-GO flakes. The decoration of TiO_2_ NRs with β-Cu_2_V_2_O_7_ NPs was obtained by an easy and fast aerosol assisted spray pyrolysis (AASP) deposition technique. A further improvement of the performances was obtained by the addition of partially reduced graphene oxide (r-GO), a p-type semiconductor [20], to reduce the charge transfer resistance. We show here that GO, deposited by electrophoretic deposition, can efficiently coat the surface of the β-Cu_2_V_2_O_7_/TiO_2_ nanostructures and, after a mild annealing, is transformed into r-GO causing a remarkable enhancement of the photocurrent with increased durability (from 50 µA/cm^2^ in the case of pure TiO_2_ NRs, 150 µA/cm^2^ for TiO_2_NRs decorated with β-Cu_2_V_2_O_7_ NP to 250 µA/cm^2^ for r-GO/β-Cu_2_V_2_O_7_/TiO_2_).

The prepared films were characterized by wide angle X-ray diffraction (WAXD), scanning electron microscopy (SEM), high resolution transmission electron microscopy (HR-TEM), UV–Vis and Raman spectroscopy, Electrochemical Impedance Spectroscopy (EIS) and PEC measurements. Their surface composition was also studied by X-ray Photoemission Spectroscopy (XPS) before and after the PEC work, under illumination, with the intention of verifying V loss and concomitant formation of CuO*_x_* passivating layers.

## 2. Materials and Methods

### 2.1. Material Preparation

All the reagents used in this study were analytical grade and purchased from Sigma-Aldrich (Milan, Italy). The photoactive material of this study, β-Cu_2_V_2_O_7_ was deposited on FTO or FTO/TiO_2_ substrates by a quick and convenient AASP method. This method consisted of the evaporation of a precursor solution micro-droplets onto a heated substrate. The micro-droplets were produced by the nebulization created through ultrasounds. Using a stream of gas (i.e., air), the micro droplets were transported near the substrate. Malachite [Cu_2_(OH)_2_CO_3_] was used as a copper-source for the aerosol solution because it contains “clean” anionic groups (carbonate and hydroxide) that do not introduce other contamination into the solution. For the preparation of Cu_2_(OH)_2_CO_3_, potassium hydrogen carbonate (10.0 g, 100 mmol) and copper sulphate pentahydrate (10.0 g, 40 mmol) were dissolved in 150 mL of hot water within two beakers. The two solutions were mixed together after being cooled. Immediately, a teal blue precipitate formed. These precipitates were recovered by “Büchner” filtration and washed with water and ethanol. The reaction that takes place is:

2CuSO_4_(aq) + 4KHCO_3_(aq) + H_2_O(l) → Cu_2_(OH)_2_CO_3_(s) + 2K_2_SO_4_(aq) + 3CO_2_ (g)

The solid was dried on a hot plate at ca. 200 °C; this was because at a temperatures close to 300 °C, malachite starts to decompose [21]. The aerosol precursor solution was prepared using a malachite suspension in water followed by the addition of a suitable ligand to complex Cu^2+^ ions, thus preventing the direct precipitation of copper vanadate, once the vanadate source is added. After complete dissolution of the malachite suspension, the vanadium-source (NH_4_VO_3_) was added. Ammonia, citric acid (CA) and ethylenediamine (EN) were used as ligands to prepare precursor solutions 1, 2 and 3, respectively.

**Solution 1** Cu_2_(OH)_2_CO_3_ (0.23 g, 1 mmol) was added to 10 mL of deionized water followed by the addition of 2 mL of concentrated (33%) NH_3_ solution, under vigorous stirring. Ammonium vanadate, NH_4_VO_3_ (0.25 g, 2 mmol), was added until a clear solution was formed.

**Solution 2** CA (0.63 g, 3 mmol) and malachite (0.23 g, 1 mmol) were dissolved in 10 mL of deionized water. Ammonium vanadate (0.25 g, 2 mmol) was added to this solution.

**Solution 3** EN (150 µL, 2 mmol) and, malachite (0.23 g, 1 mmol) were dissolved in 10 mL of deionized water. Ammonium vanadate (0.25 g, 2 mmol) was added to this solution.

TiO_2_ NRs were fabricated on FTO glass (TiO_2_/FTO) by the hydrothermal method [22]. 2 FTO slides (1 cm × 1.5 cm × 0.2 cm) were cleaned with isopropanol and deionized water in a sonicator for 30 min. The precursor solution was prepared by combining 3.44 mL of deionized water with 3.44 mL of HCl 37% and mixing for 10 min. Finally, 120 μL of titanium isopropoxide [Ti(OCH(CH_3_)_2_)_4_] were added under vigorous stirring. This solution was poured into the Teflon-liner with the FTO substrates together. The whole system was heated to 150 °C for 4 h and cooled down to room temperature (RT), after extraction from the oven. The FTO slides were finally rinsed with abundant deionized water.

Deposition of copper vanadate, either on clean FTO or on TiO_2_ NRs, was carried out using a commercial aerosol medical device (Artsana Projet). To avoid ammonia evaporation, in the case of solution 1, an excess of ammonia water solution was used (4 mL of solution 1 and 2 mL of 33% ammonia water solution).

The aerosol was conveyed through a tube (flow rate of about 60 mL/min) to a funnel neck, just above the substrate that was positioned on a metal plate heated by a Boraelectric heater (Tectra, GmbH, Frankfurt, Germany), connected to a power supply. A K-type thermocouple was positioned between the plate and the heater, to have accurate control of the sample temperature. The substrate was heated for 20 min to 340 °C. During the deposition the temperature decreased to 320 °C. The optimal deposition time on FTO was found to be 5 min, while on TiO_2_/FTO, the best results were obtained after 3 min. The TiO_2_/FTO and the β-Cu_2_V_2_O_7_/FTO samples were then annealed in air at 450 °C for 2 and 4 h, respectively.

GO was synthetized from graphite using an Improved Hummers’ method [23], developed from Marcano et al.; GO water suspension (2.5 mg/mL; pH = 6.5) was prepared using a sonicator to disperse the flakes.

The deposition was carried out by an electrophoretic process (2.5 mg/mL GO water suspension, pH = 6.5). A 5V potential was applied for 30 s between the sample (positive pole) and a clean FTO glass. The FTO slides were separated by a distance of 1.5 cm. After the deposition, the sample was annealed in air at 200 °C for 15 min.

### 2.2. Material Characterization

The morphology and nanostructure of all samples were characterized by field-emission gun SEM (Zeiss Supra 35VP, Zeiss, Jena, Germany) and High-Resolution TEM (JEOL-2011, JEOL Ltd., Tokyo, Japan). Surface composition was determined by XPS measurements performed on a custom-built UHV chamber (base pressure = 5 × 10^−10^ mbar) equipped with a non-monochromatized double-anode X-ray source (Omicron DAR-400, Scienta-Omicron GmbH, Uppsala, Sweden), a hemispherical electron analyzer (Omicron EA-125, Scienta-Omicron GmbH, Uppsala, Sweden) and a 5-channeltrons detection assembly. The electron analyzer had an acceptance angle of ±4° and the diameter of the analyzed area was 3 mm. The spectra were acquired with Al-Kα radiation. WAXD patterns were recorded in the diffraction angular range 10−50° 2θ by a Philips X’Pert PRO diffractometer, working in the reflection geometry and equipped with a graphite monochromator on the diffracted beam (CuKα radiation, Pananlytical, Almelo, The Netherlands). Raman spectra were acquired with a Thermo-Fisher DXR Raman microscope using a 532 nm laser (5 mW), focused on the sample with a 50× objective (Thermo-Fisher Scientific, Madison, WI, USA) obtaining a spot size of about 1 μm. UV–Vis spectra were acquired in absorbance and reflectance mode on a UV–Vis Cary 5E spectrophotometer.

All the electrochemical measurements were obtained in a Na borate buffer solution prepared adding NaOH to a 0.4 M solution of boric acid until pH = 9.2 was reached (example of PEC measurement obtained in Na-sulphate solution reported in Figures S1 and S2). The measurements were made in a Teflon PEC cell (see Figure S3). A Pt wire and Ag/AgCl electrode were used as counter electrode and reference electrode, respectively. PEC measurements were obtained by a visible light emitting diode (LED) source (see Figure S4) controlled by the optical bench (Metrohm-Autolab) coupled to the Autolab PGSTAT204 (Metrohm, Utrecht, The Netherlands) instrument. The samples were mounted outside the cell and kept in position by an O-ring seal. All samples were illuminated from the back side (comparison between front-side and back-side illumination reported in Figure S5) and the electrical contact was obtained by a Cu strip attached to the FTO glass surface by Silver Conductive Paint (RS). EIS data were obtained under illumination and in the dark at 1.75 V vs. Reversible Hydrogen Electrode (RHE). The amplitude for EIS measurements was ±10 mV with the frequency range set from 10^5^ to 10^−1^ Hz, performing 50 points with logarithmic distribution. Oxygen measurement was carried out by NEOFOX-KIT PROBE from Ocean Optics (Ocean Optics, 8060 Bryan Dairy Rd, Largo, FL 33777, USA).

## 3. Results and Discussion

The PEC measurements on β-Cu_2_V_2_O_7_ films deposited on FTO (β-Cu_2_V_2_O_7_) using different aerosol solutions (solution 1–3) are discussed later in the text, nevertheless, it is useful to anticipate that vanadates prepared using solution 1 (NH_3_) gave the best PEC results with respect to the other two solutions. For this reason, only β-Cu_2_V_2_O_7_ on TiO_2_ NRs (β-Cu_2_V_2_O_7_/TiO_2_) and r-GO/β-Cu_2_V_2_O_7_ on TiO_2_ (r-GO/β-Cu_2_V_2_O_7_/TiO_2_) samples, obtained with solution 1 (see experimental section) are herein discussed. We attributed this behavior to a lower carbon contamination.

**Figure 1:**
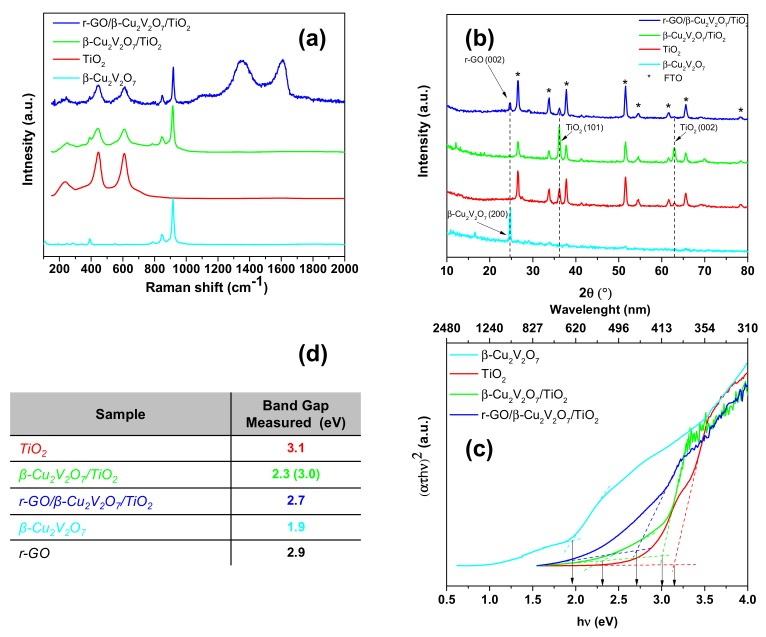
Raman spectra (**a**); wide angle X-ray diffraction (WAXD) patterns (**b**); and Tauc plots (**c**) of β-Cu_2_V_2_O_7_, TiO_2_ NRs, β-Cu_2_V_2_O_7_/TiO_2_ and GO/β-Cu_2_V_2_O_7_/TiO_2_; Bandgap values obtained from Tauc plots are indicated in the table on the left (**d**).

Figure 1a shows the Raman spectra of β-Cu_2_V_2_O_7_, TiO_2_, β-Cu_2_V_2_O_7_/TiO_2_ and r-GO/β-Cu_2_V_2_O_7_/TiO_2_, measured at room-temperature. The Raman region of pure TiO_2_ NRs presents all the characteristic peaks corresponding to rutile, that is, the peak at 244 cm^−1^ corresponding to the phonon scattering mode of rutile, the signal at 438 cm^−1^ assigned to the *E*_g_ mode, and the peak at 621 cm^−1^ to the A_1g_ mode [24]. The peak centered at 914 cm^−1^ is the characteristic band assigned to the β-Cu_2_V_2_O_7_ (VO_3_ stretching mode) [25]. In the case of r-GO/ β-Cu_2_V_2_O_7_/TiO_2_ NRs sample, two broad peaks at 1354 (*I*_D_) and 1598 cm^−1^ (*I*_G_) are those characteristic of r-GO [26]. In particular, the *I*_D_/*I*_G_ ratio corresponding to thick r-GO flakes was 0.97, as reported in reference [26], while in the case of areas where the r-GO coating was not visible by the micro-Raman microscope (50×), the ratio was 0.89 before PEC measurements and was reduced to 0.85 after PEC measurements (see Supplementary Material, Figure S6). These values are completely consistent with the presence of r-GO. Figure 1b shows the WAXD patterns of the prepared samples: β-Cu_2_V_2_O_7_, β-Cu_2_V_2_O_7_/TiO_2_, and r-GO/β-Cu_2_V_2_O_7_/TiO_2_. The diffraction peak at 2θ = 24.7° (cyan curve) is assigned to reflections from planes (200) of monoclinic β-Cu_2_V_2_O_7_ (JCPDS No. 73-1032), while peaks at 2θ = 36.2° and 62.9° (red, green and blue curves) correspond to reflections from planes (101) and (002) of rutile (JCPDS No. 21-1276). We calculated the lattice parameters of TiO_2_-rutile NRs before and after the coating with Cu_2_V_2_O_7_. These parameters are: *a* = 4.569(9) Å, *c* = 2.955(2) Å, remaining unchanged after the coating. The blue curve (r-GO/β-Cu_2_V_2_O_7_/TiO_2_) presents a further diffraction peak, at 2θ =24.7°, that has to be attributed to r-GO [27].

Indeed, the XRD pattern of β-Cu_2_V_2_O_7_/TiO_2_ (green curve) shows an extremely weak peak at 2θ = 24.7°. However, the very low intensity of this peak, probably due to the small thickness of the β-Cu_2_V_2_O_7_ coating made it difficult to detect β-Cu_2_V_2_O_7_ by X-ray diffraction and, therefore, the presence of the reflection at 2θ = 24.7°, in the case of the r-GO/β-Cu_2_V_2_O_7_/TiO_2_ has to be related to r-GO.

The band gap values (*E*_g_) of these semiconducting materials can be estimated from the Tauc plots (Figure 1c). The absorption coefficient is calculated from Equation (1):

(1) $$C\alpha \tau =-\ln\left(\frac{T}{1-R_{\mathrm{ref}}}\right)$$

where α is the absorption coefficient, τ is the thickness of the film, C is a constant, *T* the transmittance, and *R*_ref_ the reflectance. Since all the samples studied were rather opaque it was necessary to acquire both the diffuse reflectance spectra and the transmittance spectra. The band gap (*E*_g_) was estimated by calculating the intercept of an extrapolated linear fit of the experimental data, [ατhν]^2^, to the flat portion of the plot, where no absorption occurs. The measured values of *E*_g_ for a direct transition are shown in Figure 1d [28]. β-Cu_2_V_2_O_7_ and TiO_2_ show *E*_g_ values of 1.9 and 3.1 eV, respectively. Samples β-Cu_2_V_2_O_7_/TiO_2_ and r-GO/β-Cu_2_V_2_O_7_/TiO_2_ present a Tauc plot characterized by a shape typical of composite materials [11] with intercepts at ca. 3 eV (β-Cu_2_V_2_O_7_/TiO_2_ presents an additional band gap at 2.3 eV related to copper vanadate particles) and 2.7 eV, after addition of r-GO (blue curve).

**Figure 2:**
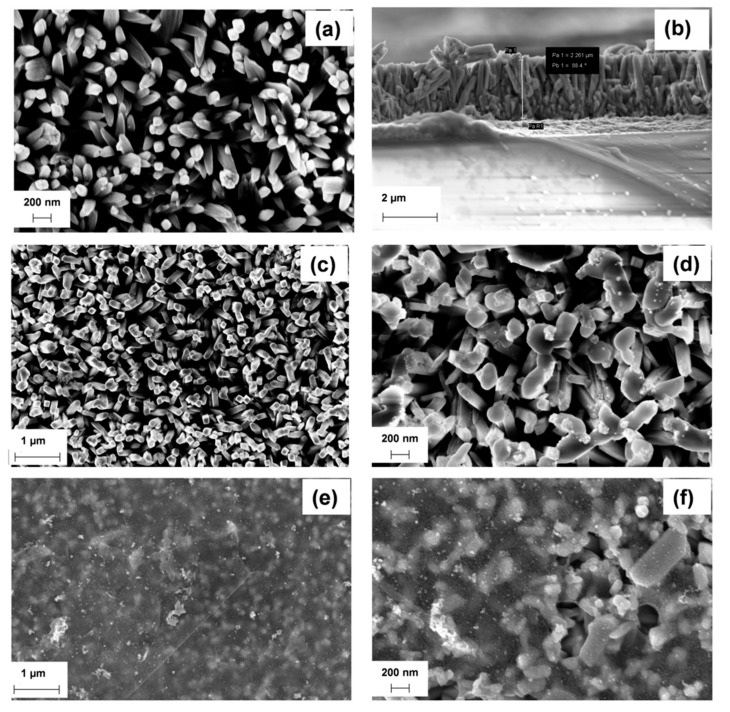
Scanning electron microscope (SEM) images of: TiO_2_ nanorods (NRs) on fluorine doped tin oxide (FTO) (**a**); cross-section of TiO_2_ NRs (**b**); β-Cu_2_V_2_O_7_/TiO_2_ (**c**,**d**); r-GO/β-Cu_2_V_2_O_7_/TiO_2_ (**e**,**f**).

Figure 2 shows the SEM images of the pure TiO_2_ NRs supported on FTO (Figure 2a) and those decorated with β-Cu_2_V_2_O_7_ (Figure 2c,d) and coated with r-GO flakes (Figure 2e,f). The as annealed film consists of TiO_2_ NRs with a diameter of ~50 nm and a length of ~2 μm (Figure 2b). The sectional view, reported in Figure 2b, shows that these TiO_2_ NRs are vertically aligned on the FTO substrate with a thickness of about 2–2.4 µm. After the deposition of β-Cu_2_V_2_O_7_, the oxide nanoparticles stick randomly on the top of TiO_2_ NRs surface (Figure 2c,d). Finally, the r-GO flakes tile the nanorods, similar to a silk coat. (Figure 2e,f).

**Figure 3:**
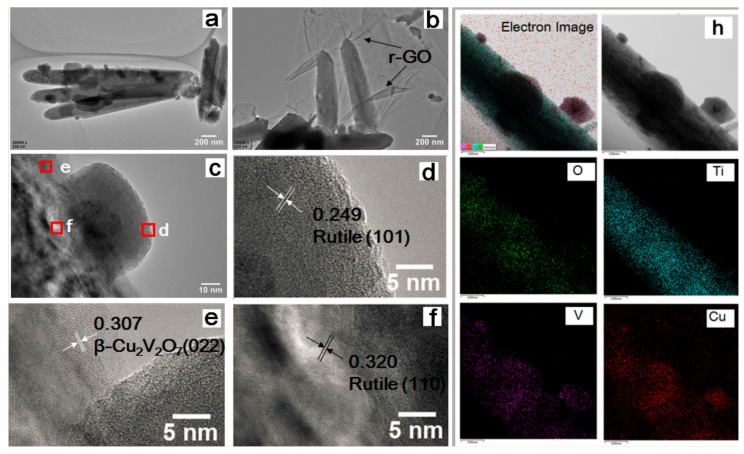
Transmission Electron Microscopy (TEM) images of β-Cu_2_V_2_O_7_/TiO_2_ (**a**); and r-GO/β-Cu_2_V_2_O_7_/TiO_2_ (**b**); High Resolution Transmission Electron Microscopy (HR-TEM) images of GO/β-Cu_2_V_2_O_7_/TiO_2_ (**c**–**f**); morphology and energy dispersive X-ray (EDX) elemental mapping of r-GO/β-Cu_2_V_2_O_7_/TiO_2_ NRs sample (**h**).

The TEM images and the corresponding energy dispersive X-ray (EDX) images are also presented in Figure 3. The size of the β-Cu_2_V_2_O_7_ NPs is between 100 and 200 nm with a regular cubic shape (Figure 3a–c). According to the measurement of the lattice fringes (*d* = 0.249, 0.320, and 0.307 nm) there is a very good match with the crystallographic planes of rutile (101), rutile (110) and β-Cu_2_V_2_O_7_ (022), respectively (Figure 3d–f). The O, Ti, V and Cu EDX elemental maps are also reported in Figure 3h together with the physical images. These images show that V and Cu are not only present on the vanadate NPs, but also on the surface of the NRs. The AASP deposition procedure allows the deposition of β-Cu_2_V_2_O_7_ crystals not only on top of the rods, but also along their length, with variable dimensions caused by the diffusion of aerosol droplets through the porous TiO_2_ NRs layer.

**Figure 4:**
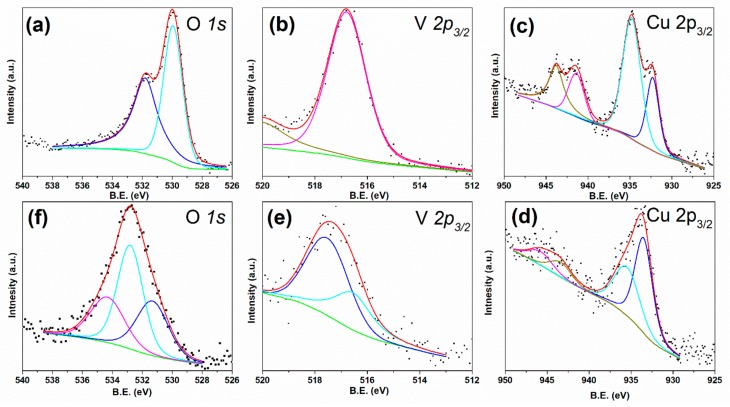
Spectroscopy (XPS) spectra of as-prepared β-Cu_2_V_2_O_7_/TiO_2_ (**a**–**c**); and r-GO/β-Cu_2_V_2_O_7_/TiO_2_ (**d**–**f**) samples.

To obtain further information on the surface composition of these nanostructures the samples were characterized by XPS, before PEC measurements, as reported in Figure 4. Figure 4 shows the O 1s, V 2p_3/2_ and Cu 2p_3/2_ XPS spectra obtained from β-Cu_2_V_2_O_7_/TiO_2_ (Figure 4a–c) and r-GO/β-Cu_2_V_2_O_7_/TiO_2_ (Figure 4d–f). The O 1s XPS spectrum of β-Cu_2_V_2_O_7_/TiO_2_ (Figure 4a) can be fitted with two components, located at about 529.8 and 532.0 eV, corresponding to lattice O^2−^ ions from metal oxides and hydroxyl groups. In the case of the sample decorated with r-GO, the O 1s signal is mainly due to the oxygen atoms bound to carbon (Figure 4f) and can be fitted with three components at 531.0, 533.0 and 534.5 eV. These three components are due to (O=C) groups, alcoholic groups (HO–C) and water, respectively. The signal at about 529.9 eV, assigned to TiO_2_ and vanadate lattice oxygens, is highly attenuated by the GO layers that coat the TiO_2_ NRs (see SEM images) [29]. In the case of copper vanadate supported on TiO_2_ NRs, without r-GO, the V 2p_3/2_ signal (Figure 4b) can be fitted with only one component at 516.8 eV with a full width at half maximum (FWHM) of about 1.5 eV, corresponding to V^5+^, while in the case of the sample decorated with r-GO, Figure 4e, the signal contains two components at 516.4 and 517.5 eV corresponding to V^4+^ and V^5+^, respectively [6]. It is interesting to note that the Cu 2p_3/2_ signal (Figure 4c,d) indicates the presence of Cu^+2^, assigned to the component at 535.4 eV, and Cu^+^ at 533.0 eV [30]. The Cu^+^ signal, in the case of the sample treated with r-GO, is actually the main component (Cu^2+^ 41% and Cu^+^ 59%), indicating that some reaction has occurred between β-Cu_2_V_2_O_7_ and GO. This is confirmed also by the presence of a quite high amount of V^4+^ (V^5+^ is 62% and V^4+^ is 38%) signal, while the Cu^2+^/V^5+^ ratio (58% Cu^2+^ and 42% V^5+^) is not too far from the 1:1 expected value for β-Cu_2_V_2_O_7_. In the case of the β-Cu_2_V_2_O_7_/TiO_2_ sample, the obtained Cu^2+^/V^5+^ ratio is also close to the expected value (40% of Cu and 60% of V) and the presence of Cu^+^ (Cu^+^ 35.5%, Cu^2+^ 64.5%) can be due to a photoreduction effect due to the X-ray source or to the presence of traces of CuO*_x_* [6].

**Figure 5:**
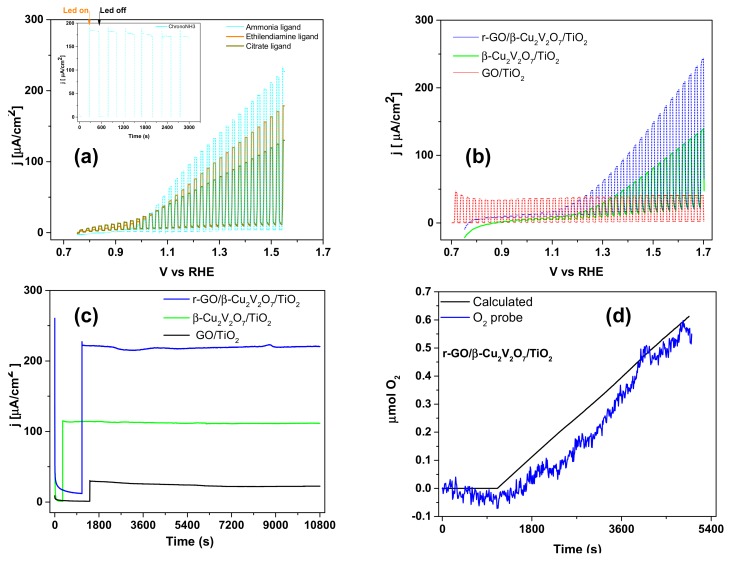
Photoelectrochemical performances: chopped Linear Sweep Voltammetry (LSV) (Borate Buffer pH = 9.2, scan rate 5 mV/s) of β-Cu_2_V_2_O_7_ deposited from aerosol solutions containing NH_3_, EN or CA, as ligands, on FTO (**a**) (the inset shows a chronoamperometry at 1.5 V vs. Reversible Hydrogen Electrode (RHE) of a sample deposited with ammonia as ligand); chopped LSV (Borate Buffer pH 9.2, scan rate 5 mV/s) of samples deposited on TiO2 NRs (**b**); chronoamperometry of β-Cu_2_V_2_O_7_/TiO_2_, r-GO/β-Cu_2_V_2_O_7_/TiO_2_ and r-GO/TiO_2_ at 1.5 V vs. RHE (**c**); comparison of calculated and measured O_2_ when r-GO/β-Cu_2_V_2_O_7_/TiO_2_ is used as working electrode with light is set on at ca. 1000 s (**d**).

All substrates were tested in PEC experiments, where the light source was a neutral white led with intensity ca. 100 mW/cm^2^ (Figure S4) in Na-borate buffer electrolyte (pH = 9.2). In Figure 5a, we report the linear voltammetry scans under chopped light for pure β-Cu_2_V_2_O_7_ deposits obtained using different ligands. From the plot it is easy see that NH_3_ furnishes the better results in terms of photocurrent (ca. 220 µA/cm^2^ a 1.55 V vs. RHE). For this reason, the decoration of TiO_2_ NRs by β-Cu_2_V_2_O_7_ was obtained by using NH_3_ in the precursor solution. As clearly visible in Figure 5b,c, the TiO_2_ NRs decorated with β-Cu_2_V_2_O_7_ show a better performance in terms of durability with almost no variation in the photocurrent after 3 h of EC work. On the contrary, the photocurrent density is lower with respect to the pure, β-Cu_2_V_2_O_7_ on FTO (Figure 5a).

**Figure 6:**
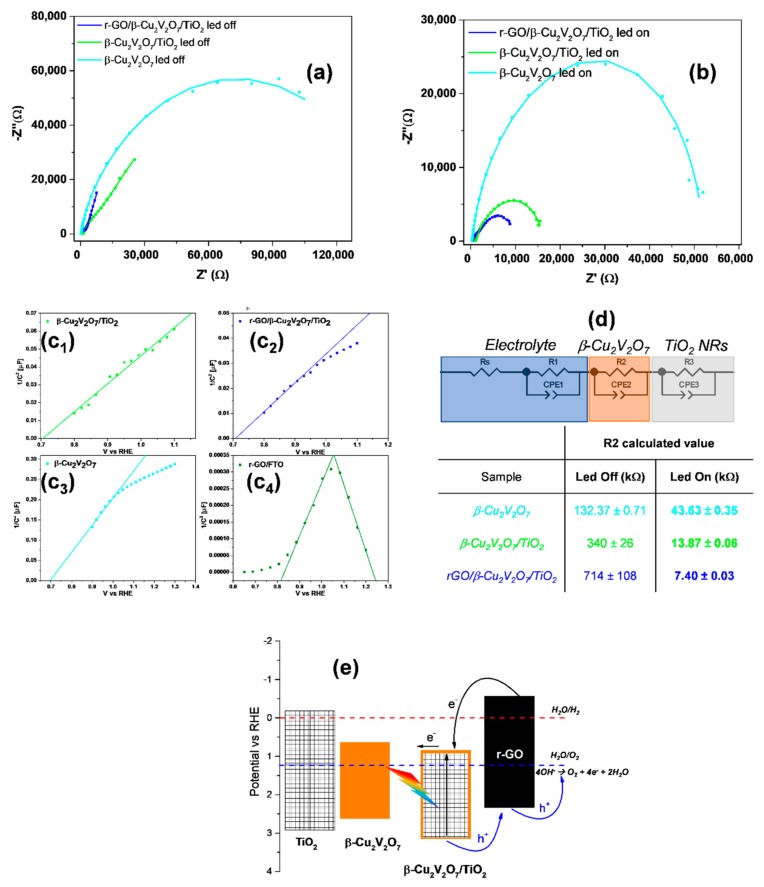
Nyquist plots obtained with samples polarized at 1.5 V vs RHE in the dark (**a**); and under illumination (**b**); Mott-Schottky plots for β-Cu_2_V_2_O_7_/TiO_2_ (**c_1_**), r-GO/ β-Cu_2_V_2_O_7_/TiO_2_ (**c_2_**), β-Cu_2_V_2_O_7_ (**c_3_**), r-GO/FTO (**c_4_**); EIS equivalent circuit [31] (**d**); schematic representation of band edges approximate position for r-GO/β-Cu_2_V_2_O_7_/TiO_2_ sample (**e**) Band edges positions for β-Cu_2_V_2_O_7_ and TiO_2_ are added for comparison.

Addition of GO flakes by electrophoretic deposition allowed to obtain a much higher photocurrent density (see Figure 5b,c) and a very good durability. Finally, in Figure 5d, we report a comparison between the O_2_ measured for the r-GO/β-Cu_2_V_2_O_7_/TiO_2_ sample in the gas phase (head-space in a sealed electrochemical cell previously purged with N_2_), by an O_2_ probe, based on the quenching of fluorescence, and the theoretical one, calculated from the recorded photocurrent. This measurement clearly demonstrates that the recorded photocurrent is not due to side processes like r-GO oxidation. The samples were also characterized by impedance spectroscopy (EIS) in the dark and under illumination at 1.5 V vs. RHE. From the data reported in Figure 6a,b it is evident that the samples β-Cu_2_V_2_O_7_/TiO_2_ and, especially r-GO/β-Cu_2_V_2_O_7_/TiO_2_, are characterized by a much lower charge transfer resistance. The equivalent circuit used to fit the data [31] contains 2 RQ elements (parallel connection of an ohmic resistance R and a constant phase element Q), in the case of pure β-Cu_2_V_2_O_7_ on FTO, while for the composite materials β-Cu_2_V_2_O_7_/TiO_2_ and r-GO/β-Cu_2_V_2_O_7_/TiO_2_, we have used a series of 3 RQ elements. This circuit is represented in Figure 6d where the R_s_ represents the solution resistance, the first RQ element the double layer, the second one the Cu-vanadate layer and the third one the TiO_2_ NRs. It is interesting to point out that upon illumination only the second circuit (R_2_) shows a very strong decrease in the charge transfer resistance, while the other 2 circuits present only minor variations. This is a strong indication that it is mainly the Cu-vanadate layer that acts as the active material in the water photo-oxidation, while the role of TiO_2_ is simply that of a substrate.

In Figure 6c we report also the Mott-Schottky (MS) plots (in the range 1–10^5^ Hz) obtained from pure β-Cu_2_V_2_O_7_, β-Cu_2_V_2_O_7_/TiO_2_ and r-GO/β-Cu_2_V_2_O_7_/TiO_2_. The relation between the flat-band potential (*V*_fb_) and the material capacity (*C*) is reported in Equation (2). *N*_SC_ indicates the carrier’s concentration in the space charge of the material, *ε* the dielectric constant, *e* is the electron charge and *A* is the area of the electrode. n-types semiconductors, like TiO_2_ and β-Cu_2_V_2_O_7_, are characterized by positive slopes, while p-types materials have negative slopes.

(2) $$\frac{1}{C^{2}}=\frac{2\left(V-V_{fb}\right)}{eN_{sc}\varepsilon A^{2}}$$

The capacity values were calculated by fitting the impedance data with a Randle’s circuit containing Constant Phase Elements (CPE) instead of ideal capacitors. Thus, the capacity was calculated from Brugg’s Equation (3) [32]

(3) $$C={\left(Q\right)}^{\frac{1}{p}}{\left(R_{S}{}^{-1}+R_{p}{}^{-1}\right)}^{\left(1-\frac{1}{p}\right)}$$

where *Q* and *p*, are fitting parameters from CPE elements, *R*_s_ is the cell resistance and *R*_p_ is the resistance in parallel with CPE elements. By plotting *C*^−2^ vs. RHE it is possible to determine *V*_fb_ and from this value to derive the approximate position of conduction (CB) and valence (VB) edges. The relation between *V*_fb_ and bands edges (*E*_cb_ and *E*_vb_) can be expressed by Equations (4) and (5) [33]:

(4) $$E_{\mathrm{cb}}=\;V_{\mathrm{fb}}+\;k_{b}T\ln\left(\frac{N_{\mathrm{sc}}}{N_{\mathrm{cb}}}\right)$$

(5) $$E_{\mathrm{vb}}=-\;V_{\mathrm{fb}}+\;k_{b}T\ln\left(\frac{N_{\mathrm{sc}}}{N_{\mathrm{vb}}}\right)$$

where *N*_cb_ and *N*_vb_, are the effective density of states in the CB and VB for a n-type and p-type semiconductors, respectively. In the case of n-type conductivity Equation (4) is usually approximated with *E*_cb_ ≈ *V*_fb_ + 0.1 eV [8]. Thus, the MS plots reported in Figure 6c, show how the decoration of TiO_2_ does not change band edges position of the copper vanadate (TiO_2_ acts as a support), while the addition of p-type GO, probably produces many p-n nano-junctions with β-Cu_2_V_2_O_7_/TiO_2_ (see scheme of Figure 6d, although not visible from the MS plot of Figure 6c_2_. Indeed, the lower slope of the MS plot of Figure 6c_2_ indicates a higher concentration of electrons (1 × 10^17^ m^−3^ for β-Cu_2_V_2_O_7_/TiO_2_ and 1.4 × 10^17^ m^−3^ for the sample decorated with r-GO), as already seen in the case of TiO_2_ nanorods decorated with Cu_2_O nanoparticles [34]. Finally, the p-type conductivity of r-GO is clearly seen from the MS plot obtained from a pure r-GO sample deposited on FTO and thermally treated at 200 °C for 15 min (Figure 6c_4_).

**Figure 7:**
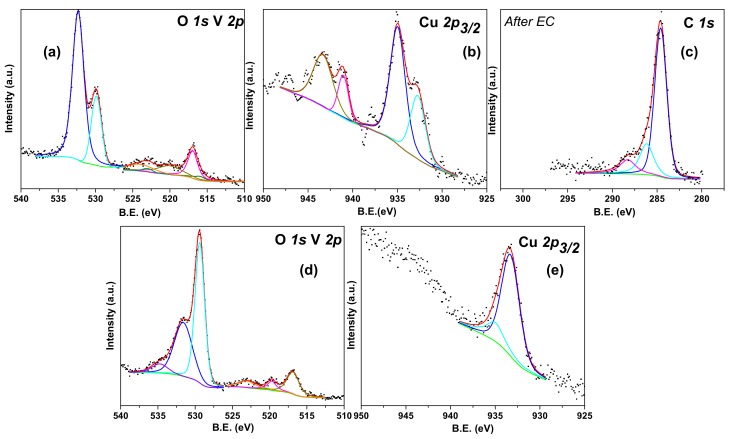
XPS spectra of samples β-Cu_2_V_2_O_7_/TiO_2_ after Electrochemical work (**a**–**b**); and of r-GO/ β-Cu_2_V_2_O_7_/TiO_2_ (**c**–**e**) after photoelectrochemical (PEC) work.

More precise details about the surface composition of these nanostructures can be obtained by acquiring XPS data after electrochemical work. The results of this analysis are summarized in Figure 7 and Table 1. Figure 7a,b shows the O 1s, V 2p and Cu 2p XPS spectra obtained from β-Cu_2_V_2_O_7_/TiO_2_ NRs after 3 h of electrochemical measurements, under illumination. The O 1s XPS spectrum can be fitted with two components located at about 529.9 and 532.0 eV that correspond to lattice O^2−^ ions from β-Cu_2_V_2_O_7_ and TiO_2_ and hydroxyls groups [35]. The V 2p_3/2_ signal (in Figure 7a), can be fitted with two components at 516.0, weak, and 517.0 eV corresponding to V^4+^ and V^5+^ respectively. The Cu 2p_3/2_ signals (Figure 7b) contains two components, one at 935.0 and another at 933.0 eV indicating the presence of Cu^2+^ and a considerable quantity of Cu^+^. The presence of Cu^+^ is probably caused by a photo-reduction effect and eventually by X-rays in UHV. In Figure 7c we show also the C 1s spectra acquired from a sample of r-GO/β-Cu_2_V_2_O_7_/TiO_2_ after PEC work. The region can be fitted with 3 components at 284.1, 285.7 and 288.0 eV corresponding respectively to C–C, C–O and C=O bonds [26]. The relative intensity and positions of these signals are fully compatible with p-type r-GO oxide, after a mild heat treatment [36]. A simple visual inspection of the O 1s and V 2p region, reported in Figure 7d, reveals how the amount of V in the case of the r-GO/β-Cu_2_V_2_O_7_/TiO_2_ is significantly lower if compared with the sample not containing GO. In fact, the V 2p signal is much lower with respect to the O 1s signal at 529.4 eV. Moreover, the Cu 2p signal, reported in Figure 7e, shows mostly the presence of Cu^+^ deduced from the position (933.0 eV) and the very low intensity of the satellites peaks. This fact is in agreement with what already verified on the sample before PEC work where the high amount of Cu^+^ and the high Cu/V ratio indicated that the addition of GO modified the composition of the vanadate.

The Cu/V ratios obtained from XPS data after EC work are similar to those obtained before EC (Raman spectra and SEM images after EC are reported in Figures S6 and S7, respectively). In the case of β-Cu_2_V_2_O_7_/TiO_2_ we found a 52% abundance of V^5+^ and 48% for Cu^2+^, with a rather high amount of Cu^+^ (Figure 7b). In the case of the sample decorated with r-GO, the amount of Cu^2+^ is 32%, while the amount of V^5+^ is 68%. In this last case the large quantity of Cu^+^ seems to be due to the presence of the GO layer.

All the above reported data indicate that supporting β-Cu_2_V_2_O_7_ on TiO_2_ NRs allows the obtainment of a material with good durability, as photoanode, with a much lower charge transfer resistance as testified by the EIS data (Figure 6a,b). The interaction between the TiO_2_ NRs and β-Cu_2_V_2_O_7_ has a favorable effect on the photocurrent production since the deposition of V_2_O_5_ on TiO_2_ NRs, by the same AASP process, does not lead to any particular enhancement in the photocurrent if compared with untreated TiO_2_ (see Figures S8 and S9). It’s also known that the decoration of Degussa P25 by CuO nanoparticles actually leads to a modest enhancement of photocurrent values [37]. A significant improvement in the photocurrent density can be achieved by decorating the β-Cu_2_V_2_O_7_/TiO_2_ sample by GO followed by heat treatment at about 200 °C. The addition of GO changes the Cu/V ratio leading to high amount of Cu^+^ as testified by the XPS spectra, acquired before and after EC work. We think that the better performance in terms of photocurrent is due to the combination of r-GO/β-Cu_2_V_2_O_7_/TiO_2_ since the addition of r-GO to TiO_2_ NRs did not lead to any particular improvement in the photocurrent, as is clearly visible in Figure 5b. We verified that the interaction of GO with β-Cu_2_V_2_O_7_ and heat treatment at 200 °C, after the electrophoretic deposition of GO, actually leads to the formation of p-type r-GO with a band gap, as measured from the UV–Vis spectra (see Figure S10) of about 2.5–2.7 eV. We can justify the interaction of GO with β-Cu_2_V_2_O_7_, that leads to the variation in the Cu/V ratio, as caused by the relatively low pH of the GO suspension (pH = 6.5), used during the electrophoretic deposition process, and by the GO itself. In fact, the solubility of β-Cu_2_V_2_O_7_ increases at low pH and, at the same time, the GO sheets can easily co-ordinate the Cu^2+^ ions [38]. The Cu^2+^ ions, once chemisorbed on the GO nano-sheets, most probably by the carboxylic groups, can be reduced by GO and most probably by the heat treatment, with formation of CO_2_ as summarized in the following reaction sequence [38]:

[Cu(H_2_O)_4_]^2+^ + GO → GO-[Cu(H_2_O)_4_]^2+^

GO-[Cu(H_2_O)_4_]^2+^ → GO-[Cu(H_2_O)_2_]^2+^

GO-[Cu(H_2_O)_2_]^2+^ + heat → r-GO-[Cu(H_2_O)_2_]^+^ + CO_2_

As known and reported in several publications, the mild thermal treatment at 200–210 °C that leads to the formation of a partially reduced GO has to be intended formally as a disproportionate reaction where the electrons released with O_2_, CO or CO_2_ evolution are used to reduce the GO surface [39,40]. In this particular case, we think that this process leads also to the formation of Cu^+^ species as Cu_2_O nanoparticles. Since the *V*_fb_ is the same for β-Cu_2_V_2_O_7_, β-Cu_2_V_2_O_7_/TiO_2_ and r-GO/β-Cu_2_V_2_O_7_/TiO_2_ and the Cu^2+^/V^5+^ ratio on r-GO/β-Cu_2_V_2_O_7_/TiO_2_ is compatible with a 1:2 value, we can formulate the hypothesis that the vanadate partially decomposes forming Cu_2_O nanoparticles on the GO flakes and on the NRs surface, while remaining still on the TiO_2_ surface, in lower amounts, in a form compatible with the a “CuV_2_O_6_” stoichiometry. The position of band edges of r-GO with respect to the band edges of the composite material, β-Cu_2_V_2_O_7_/TiO_2_, is particularly favorable to form many p-n nano-junctions, as depicted in Figure 6d, leading to a better charge separation, increase in the photocurrent density and improved durability. It is important to note that the slightly higher position of *E*_cb_ of pure TiO_2_ NRs (slightly above the H^+^/H_2_ reduction potential) would have led to a less favorable junction with p-type r-GO. Finally, the possible formation of a further p-n junction between the Cu_2_O nanoparticle and the TiO_2_ surface should also be taken into account [34].

## 4. Conclusions

We have prepared β-Cu_2_V_2_O_7_/TiO_2_ and r-GO/β-Cu_2_V_2_O_7_/TiO_2_ composite materials with the aim of obtaining visible light driven photoanodes. The vanadate deposition was obtained by an easy & fast aerosol assisted spray pyrolysis procedure. The β-Cu_2_V_2_O_7_/TiO_2_ composite material showed a better durability if compared with pure β-Cu_2_V_2_O_7_ deposited on FTO and a lower charge transfer resistance as indicated by EIS data. The addition of p-type r-GO to β-Cu_2_V_2_O_7_/TiO_2_ had a positive effect on durability, charge transfer resistance and photocurrent density. As verified by XPS analysis, the GO addition, by electrophoretic deposition, led to a strong interaction with β-Cu_2_V_2_O_7_ with formation of r-GO flakes and Cu_2_O nanoparticles. The amount of O_2_ produced, upon visible light illumination, independently measured by an O_2_ probe, indicated that this composite material is characterized by a good faradaic efficiency. The easy and fast procedure that allows its preparation can be easily extended, with low cost, to electrodes with a larger area.

## Figures and Tables

**Table 1:** Cu and V percent abundance from XPS data.

Element Abundance	Before PEC		After PEC	
Sample	Cu^2+^	V^5+^	Cu^2+^	V^5+^
r-GO/β-Cu_2_V_2_O_7_/TiO_2_	58	42	32	68
β-Cu_2_V_2_O_7_/TiO_2_	40	60	52	48

## Supplementary Materials

The following are available online at http://www.mdpi.com/2079-4991/8/7/544/s1. Figure S1. Chopped Linear Sweep Voltammetry of sample r-GO/β-Cu_2_V_2_O_7_/TiO_2_ in Na sulfate electrolyte; Figure S2. Chronoamperometry from sample r-GO/β-Cu_2_V_2_O_7_/TiO_2_ at 1.75 V vs. RHE; Figure S3. Drawing of the photoelectrochemical cell (Proteus Gamma I—PINE Research) (CE = Counter Electrode; RE = Reference Electrode); Figure S4. Emission spectrum of white LED light used in all the reported PEC measurements; Figure S5. Chopped LSV on r-GO/β-Cu_2_V_2_O_7_/TiO_2_ sample with front and back illumination (ca. 100 mW/cm^2^) in borate buffer solution; Figure S6. (a) Raman spectra before and after PEC work obtained from different areas of sample r-GO/β-Cu_2_V_2_O_7_ after (b) and before (c) PEC work; Figure S7. SEM image of sample r-GO/β-Cu_2_V_2_O_7_/TiO_2_ after PEC work; Figure S8. Chopped Linear Sweep Voltammetry of the TiO_2_ nanorods substrate in borate buffer (pH = 9.2) with led light intensity of ca. 100 mW/cm^2^; Figure S9. Chopped Linear Sweep Voltammetry of TiO_2_ NRs decorated with V_2_O_5_ nanoparticles in borate buffer (pH = 9.2); Figure S10. Tauc Plot of r-GO deposited by electrophoresis on FTO slides.
