# Supplementary material for: Visible Light Driven Photoanodes for Water Oxidation Based on Novel r-GO/β-Cu2V2O7/TiO2 Nanorods Composites
Source: Nanomaterials (Basel). 2018 Jul 18;8(7):544. doi: 10.3390/nano8070544 (PMC6070958; doi:10.3390/nano8070544)
Supplement: Supplementary file 1 [file nanomaterials-08-00544-s001.pdf]

Supplementary Material

Visible Light Driven Photoanodes for Water Oxidation based on novel r-GO/β-$Cu_2V_2O_7$/ TiO_2 nanorods composites

Shuang Shuang [2,3,§], Leonardo Girardi [1,§], Gian Andrea Rizzi [1*‡], Andrea Sartorel [1], Carla Marega [1], Zhengjun Zhang [3] and Gaetano Granozzi [1]

[1] University of Padova and INSTM Unit, via Marzolo 1, Padova 35121, Italy
[2] State Key Laboratory of New Ceramics and Fine Processing, School of Materials Science and Engineering, Tsinghua University, Beijing 100084, China
[3] Key Laboratory of Advanced Materials (MOE), School of Materials Science and Engineering, Tsinghua University, Beijing 100084, China

§ These two authors contributed equally

* Correspondence: gianandrea.rizzi@unipd.it; Tel.: +39-049-827-5722

‡ Corresponding author

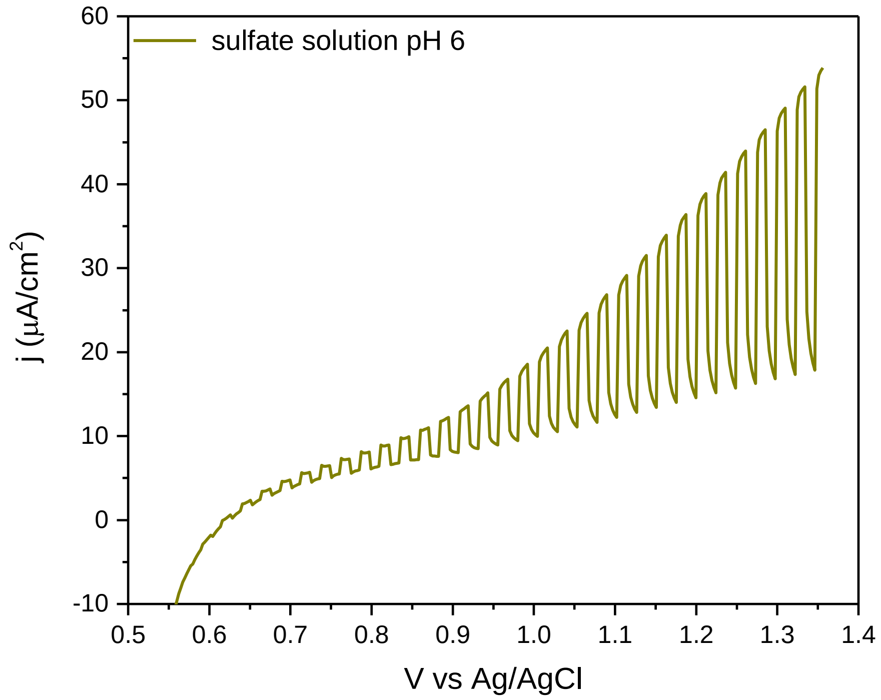

Figure S1. Chopped Linear Sweep Voltammetry of sample r-GO/β-$Cu_2V_2O_7$/TiO_2 in Na sulfate electrolyte.

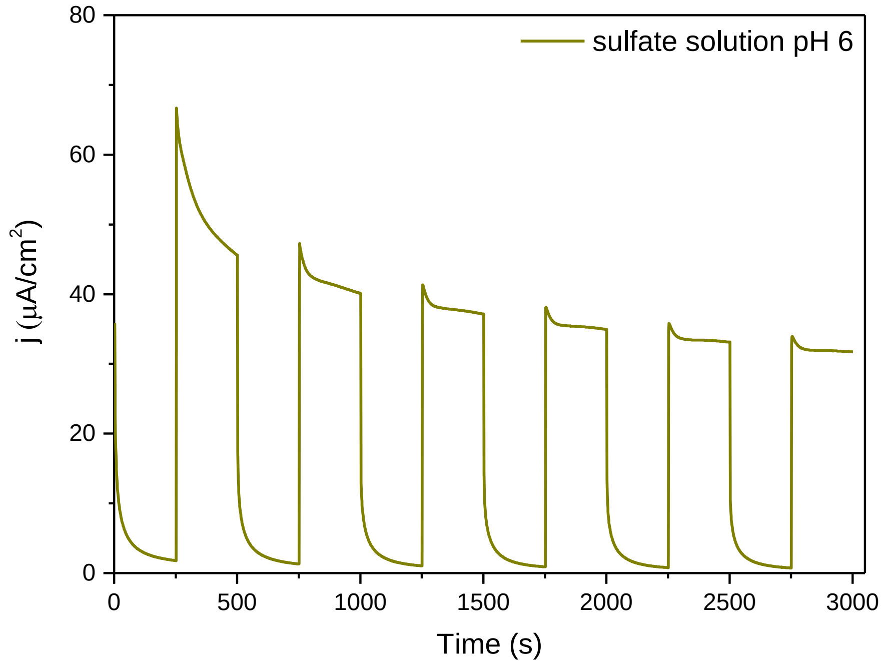

Figure S2. Chronoamperometry from sample r-GO/β-$Cu_2V_2O_7$/TiO_2 at 1.75 V vs RHE.

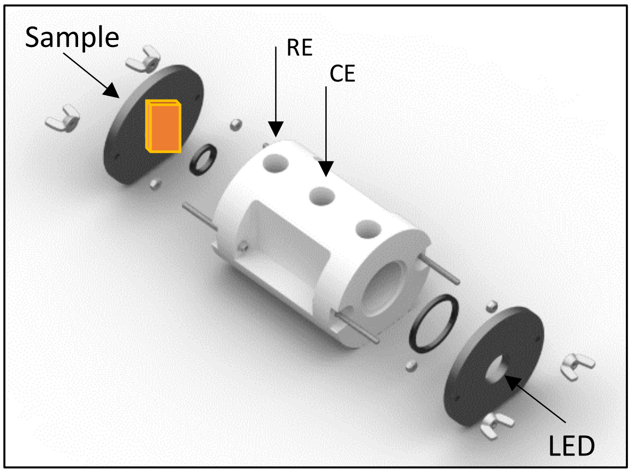

Figure S3. Drawing of the photoelectrochemical cell (**Proteus Gamma I - PINE Research**) (CE = Counter Electrode; RE = Reference Electrode).

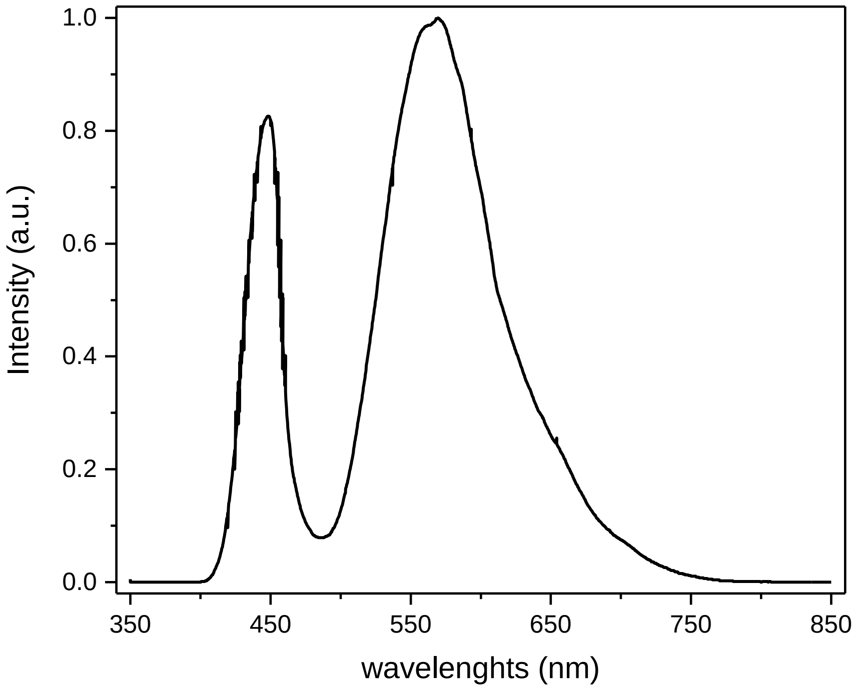

Figure S4. Emission spectrum of white LED light used in all the reported PEC measurements.

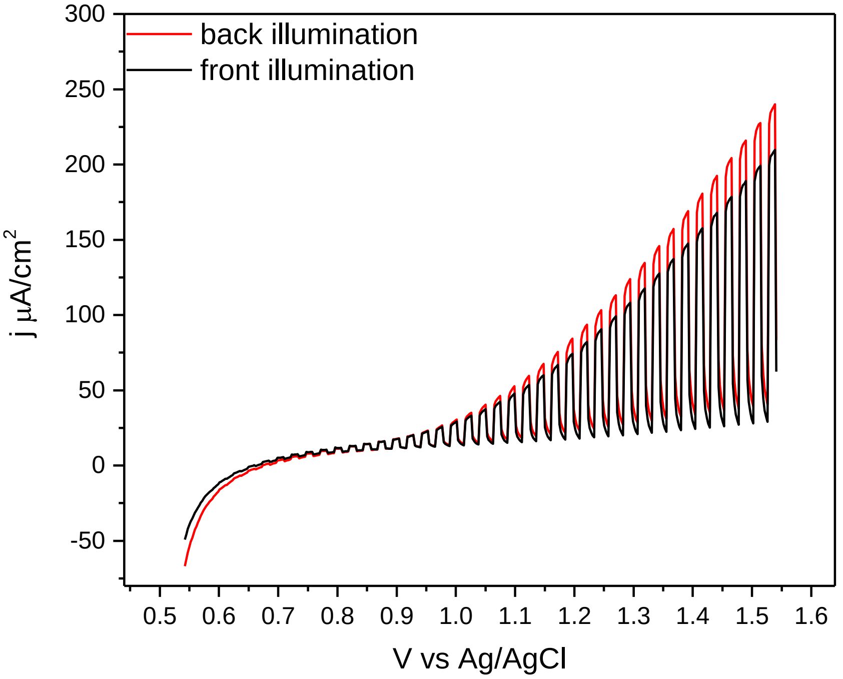

Figure S5. Chopped LSV on r-GO/β-$Cu_2V_2O_7$/TiO_2 sample with front and back illumination (ca 100mW/cm^2) in borate buffer solution.

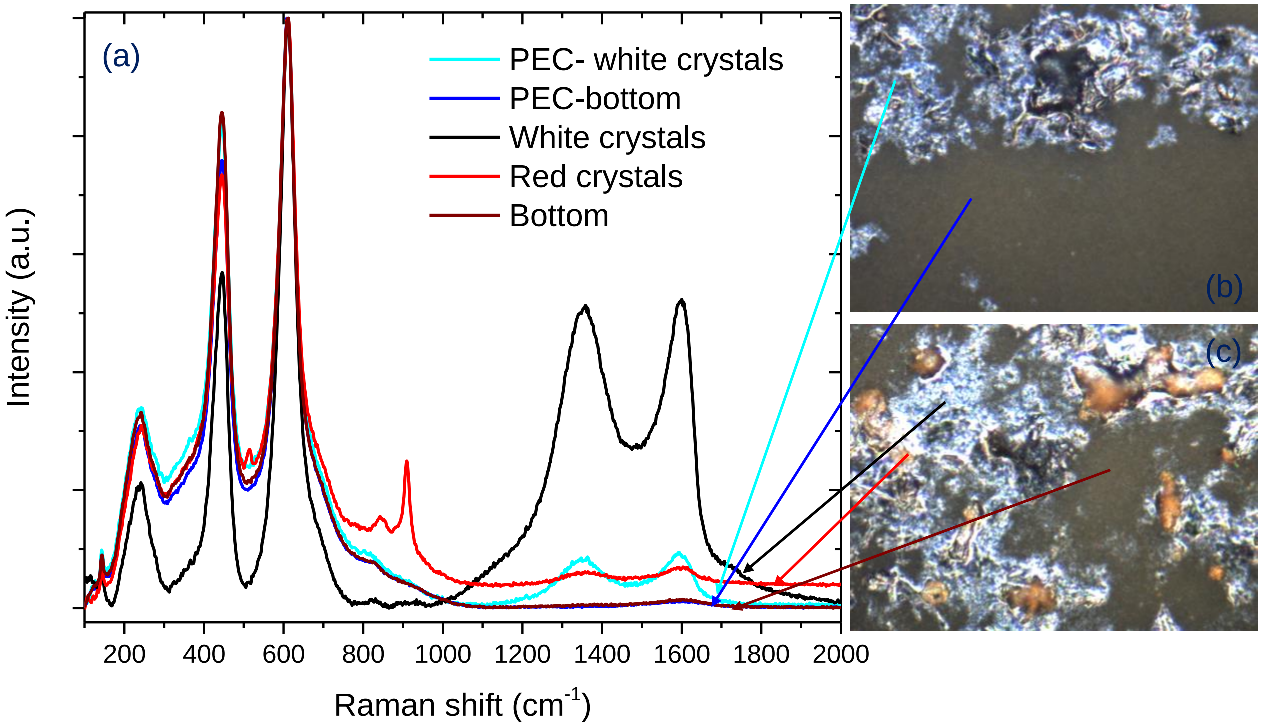

Figure S6. **(a)** Raman spectra before and after PEC work obtained from different areas of sample r-GO/β-$Cu_2V_2O_7$ after **(b)** and before **(c)** PEC work.

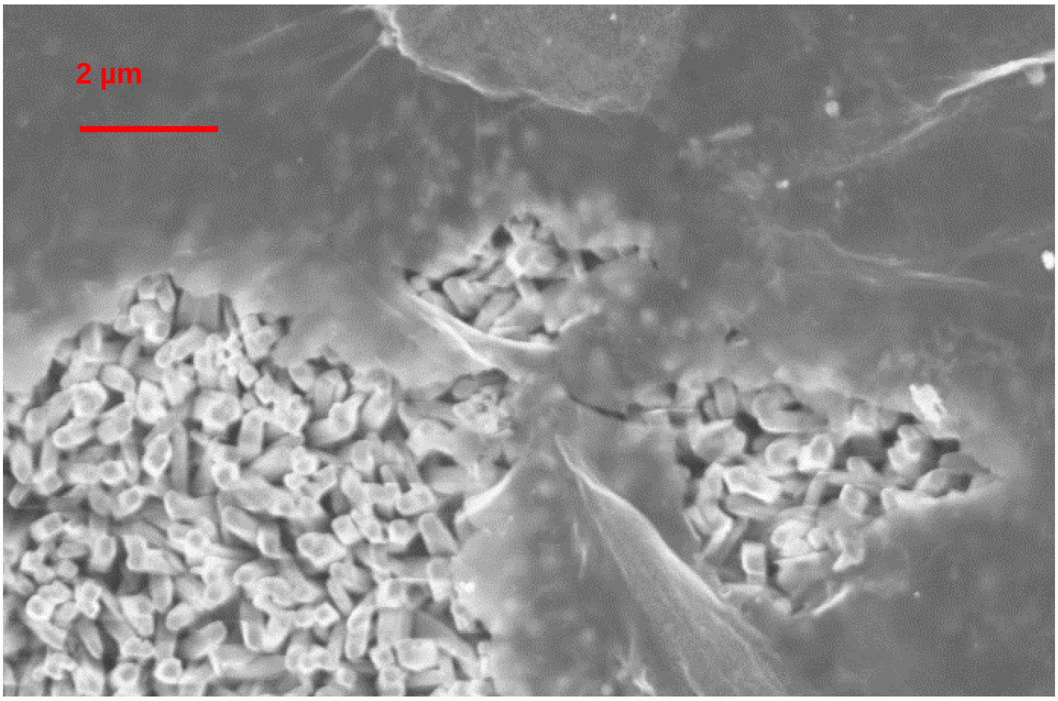

Figure S7. SEM image of sample r-GO/β-$Cu_2V_2O_7$/TiO_2 after PEC work.

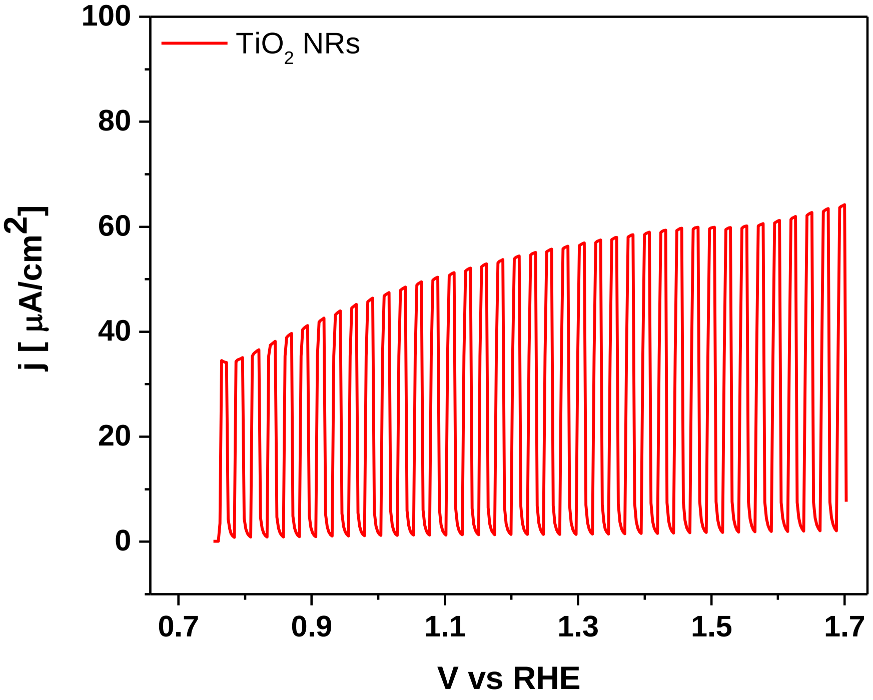

Figure S8. Chopped Linear Sweep Voltammetry of the TiO_2 nanorods substrate in borate buffer (pH 9.2) with led light intensity of c.a. 100mW/cm^2.

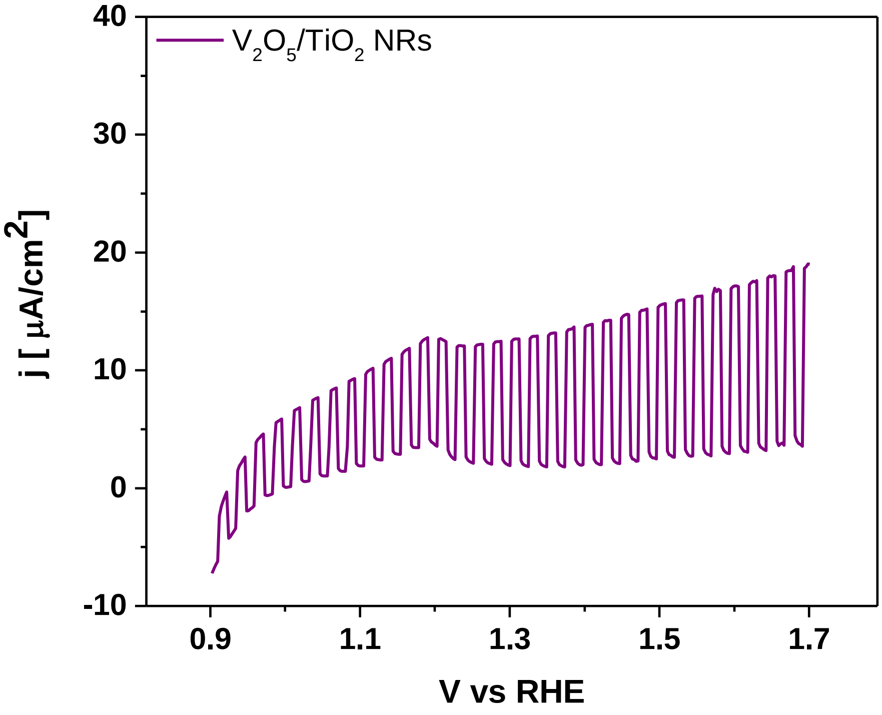

Figure S9. Chopped Linear Sweep Voltammetry of TiO_2 NRs decorated with V_2O_5 nanoparticles in borate buffer (pH 9.2)

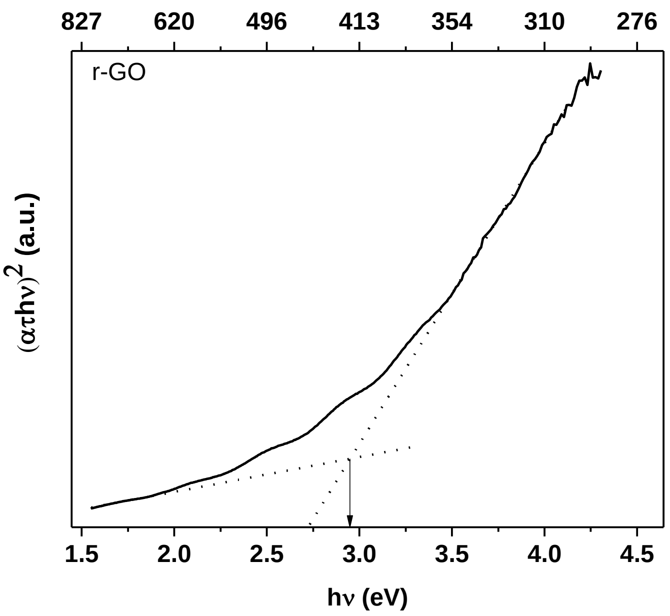

Figure S10. Tauc Plot of r-GO deposited by electrophoresis on FTO slides.
